# Regulatory sampling of industrial hemp plant samples (*Cannabis sativa* L.) using UPLC-MS/MS method for detection and quantification of twelve cannabinoids

**DOI:** 10.1186/s42238-020-00050-0

**Published:** 2020-12-10

**Authors:** Erin C. Berthold, Rui Yang, Abhisheak Sharma, Shyam H. Kamble, Siva R. Kanumuri, Tamara I. King, Raluca Popa, Joshua H. Freeman, Zachary T. Brym, Bonnie A. Avery, Christopher R. McCurdy

**Affiliations:** 1 Department of Pharmaceutics, College of Pharmacy, University of Florida, Gainesville, FL USA; 2 North Florida Research and Education Center, University of Florida, Quincy, FL USA; 3 Translational Drug Development Core, Clinical and Translational Science Institute, University of Florida, Gainesville, FL USA; 4 Tropical Research and Education Center, University of Florida, Homestead, FL USA; 5 Department of Medicinal Chemistry, College of Pharmacy, University of Florida, Gainesville, FL 32610 USA

**Keywords:** Cannabis, Liquid chromatography–mass spectroscopy, Cannabinoid assay, Hemp, Cannabidiol, Tetrahydrocannabinol

## Abstract

**Background:**

In 2018, the Farm Bill mandated the United States Department of Agriculture to develop regulations governing the cultivation, processing, and marketing of industrial hemp. Industrial hemp is defined as *Cannabis sativa* L. with a total Δ-9-tetrahydrocannabinol (Δ-9-THC) content ≤0.3%. Therefore, for hemp to become an agricultural commodity, it is important to regulate production by developing standard methods for sampling and testing of the plant material.

**Methods:**

An ultra-performance liquid chromatography-tandem mass spectrometry analytical method for the quantification of twelve cannabinoids was developed. The method was applied to a regulatory sampling trial of three hemp varieties cultivated for cannabidiol (CBD) production. Two samples were taken from 28 plants with one sample being flower only while the other was a composite sample that included flowers, leaves, and stems.

**Results:**

The assay method was validated for specificity, range, repeatability, reproducibility, and recovery in accordance with all applicable standards for analytical methods. The results of the regulatory study indicated a significant decrease in the concentration of total Δ-9-THC and total CBD of 0.09% and 1.32%, respectively, between a flower only and a composite sample.

**Conclusions:**

There are many factors that may influence reported total Δ-9-THC content in industrial hemp. A robust analytical method was developed to analyze hemp samples in a trial regulatory study. The results indicate that the way hemp is sampled and analyzed may influence the legality of a crop, which could have negative economic and legal consequences.

## Introduction

*Cannabis sativa* L. is a source of one of the oldest known drugs in the world, cannabis, and one of the oldest known crops, industrial hemp, having been found in tombs dating back to 8000 BC (Deiana et al. 2012). The biologically active compounds in the plant are called cannabinoids, of which over one hundred have been identified to date (Hanus 2009). Being morphologically and taxonomically similar, the only characteristic that legally distinguishes industrial hemp from cannabis is the concentration of the main psychoactive component, Δ-9-tetrahydrocannabinol (Δ-9-THC), in the plant.

Regulations for sampling and testing of industrial hemp to determine total THC content are being developed. Industrial hemp was removed from the statutory definition of cannabis if the total THC content does not exceed 0.3% on a dry weight basis (Agricultural Improvement Act of 2018 2018). Total THC is defined by the following formula:

$$ Total\  THC={Concentration}_{\varDelta -9- THC}+\left({Concentration}_{\varDelta -9- THC A}\ast 0.877\right) $$

Δ-9-Tetrahydrocannabinolic acid (THCA) is the molecular precursor to Δ-9-THC. When the plant material is exposed to heat, light, or alkaline conditions, THCA will convert to Δ-9-THC. Determining total THC content allows for the quantification of all potential Δ-9-THC present in plant material.

In 2019, the University of Florida’s Institute of Food and Agricultural Science (UF/IFAS) initiated cultivation studies on over 40 varieties of industrial hemp throughout the state of Florida. The first goal of this study was to develop a robust analytical method used to assess the cannabinoid content of these varieties; not only to ensure legality but also the additional ten minor cannabinoids to build a chemical fingerprint repository for each variety.

There are numerous existing methods for the detection and quantitation of cannabinoids (Gul et al. 2015, 2018; Aizpurua-Olaizola et al. 2014). Recent reviews of these methods indicated that most use either gas chromatography (GC) or liquid chromatography (LC) to separate the cannabinoids, while methods for detection include mass spectrometry, photodiode array, and ultraviolet light, among others (Nahar et al. 2020a, b; Leghissa et al. 2018; Citti et al. 2018). Here, an ultra-performance liquid chromatography-tandem mass spectrometry (UPLC-MS/MS) method was developed with a short run time of 6 min. At the outset of the development of this method, no others were available in the literature that was under 8 min and able to detect and quantify twelve cannabinoids. A faster method was necessary in order to analyze the samples from over forty varieties grown across the state of Florida as part of the UF/IFAS cultivation studies. The method simultaneously separates twelve cannabinoids and quantifies them at the level of ≤0.05% on a dry weight basis.

**Fig. 1:**
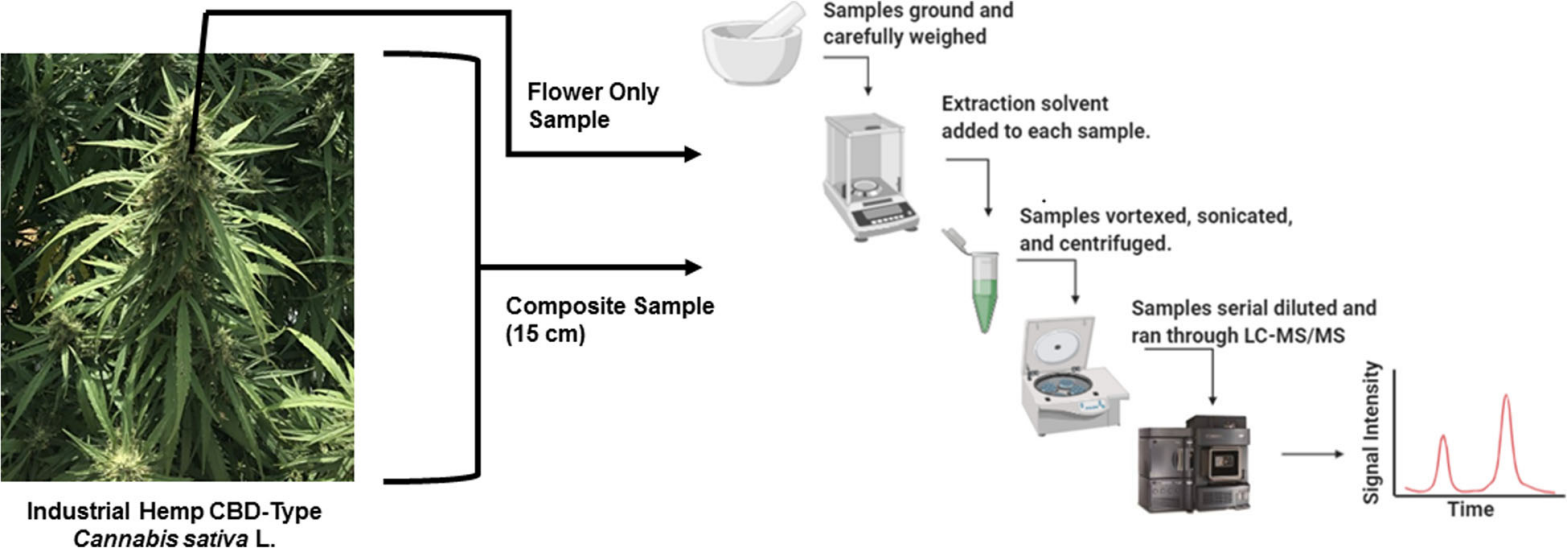
Representation of the study design. Two samples were taken from cannabidiol (CBD)-type marketed industrial hemp grown in North Florida and analyzed for cannabinoid content using a rapid, robust ultra-performance liquid chromatography-tandem mass spectrometry method

Cannabinoid concentration varies throughout the plant, with the highest concentrations in the bracts and flowers followed by significant decreases in leaves, stems, roots, and seeds (Hemphill et al. 1980; Andre et al. 2016). Currently, the Interim Final Rule for industrial hemp sampling proposed by the USDA requires inflorescent stem from the top 1/3 of the plant to be sampled, milled, and run through a screen no larger than 1.5 × 1.5 mm to remove larger twigs and stems (Establishment of a domestic hemp production program 2019). Alternatively, other draft sampling procedures recommend sampling the top 15–30 cm of the plant and grinding it down to uniform consistency prior to analysis (Hemp/CBD in Florida 2020; Guidance Procedures 2.0 2019). Since cannabinoid content varies throughout the plant, it is important to understand how the presence of leaves and stems in a sample for regulatory testing affects cannabinoid content. Therefore, the second goal was to investigate the cannabinoid content of a flower sample versus a 15-cm composite plant sample that included leaves and stems in three CBD-type varieties of day-length-sensitive marketed industrial hemp: cherry blossom (ChBL), cherry × T1 (CT1), and cherry wine (CW). To the best of our knowledge, this is the first study to look specifically at regulation lengths of hemp cuttings versus floral material to investigate the potential differences in cannabinoid content. Figure 1 demonstrates the two main goals of this study and the study design.

The results of this study will provide better insight regarding the effects of plant sampling and analysis on cannabinoid content in an effort to improve industrial hemp crop production and regulatory compliance.

## Materials and methods

### Materials and reagents

Commercially available standards (purity >98%) for cannabichromene (CBC), cannabicyclol (CBL), CBD, cannabidiolic acid (CBDA), cannabidivarin (CBDV), cannabigerol (CBG), cannabigerolic acid (CBGA), cannabinol (CBN), delta-8-tetrahydrocannabinol (Δ-8-THC), Δ-9-THC, THCA, and tetrahydrocannabivarin (THCV) were obtained from Cerilliant (Round Rock, TX, USA). Additionally, deuterated internal standards (purity >98%) (IS) delta-9-tetrahydrocannabinol-D3 (Δ-9-THC-D3) and 11-nor-9-carboxy-Δ-9-THC-D9 (11-nor-9-COOH-Δ-9-THC-D9) were also obtained from Cerilliant (Round Rock, TX, USA). LC-MS grade water, methanol, and formic acid were sourced from Fisher Scientific (Fair Lawn, NJ, USA). Commercially available hops, *Humulus lupulus*, were obtained from BioKoma (Old Mill Creek, IL, USA).

### Instrumentation and analytical conditions

An analytical method for quantification of cannabinoids was developed using a Waters I-Class Acquity UPLC coupled with a Waters Xevo TQ-S Micro™ triple-quadrupole mass spectrometer (MS/MS) (Milford, MA, USA). The analytes were separated on a Waters Acquity UPLC BEH C_18_ column (2.1 × 100 mm, 1.7 μm) using a gradient elution over 6 min (Milford, MA, USA). The mobile phase was composed of water containing 0.1% formic acid (A) and methanol and acetonitrile (50:50, v/v) (B) and set at a flow rate of 0.35 mL/min. Initial conditions were 11% A and 89% B which was held for 30 s, then linearly increased to 100% B until 5.5 min, then sharply decreased back to the initial conditions for the final 30 s to re-equilibrate the column. The weak needle wash was composed of methanol, acetonitrile, and water (1:1:2, v/v) acidified with 0.5% formic acid, while the strong needle wash was composed of methanol, acetonitrile, water, and isopropyl alcohol (1:1:1:1, v/v) acidified with 0.1% formic acid. Both wash volumes were 800 μL. The injection volume was set to 2 μL with partial needle loop overflow (to a total of 10 μL). The column oven temperature was set to 40 °C, and the autosampler temperature was set to 10 °C. Multiple reaction monitoring in positive electrospray ionization (ESI^+^) was used for neutral cannabinoids (CBC, CBL, CBD, CBDV, CBG, CBN, Δ-8-THC, Δ-9-THC, and IS Δ-9-THC-D3) while negative electrospray ionization (ESI^-^) was used for acidic cannabinoids (CBDA, CBGA, and THCA with IS 11-nor-9-COOH-Δ-9-THC-D9). The mass spectrometer settings were optimized using the IntelliStart™ feature of MassLynx® Version 4.2 (Waters, Milford, MA, USA) and transitions for each compound were selected based on which had the highest stability and abundance. The monitored transitions and instrument conditions can be seen in Table 1.

**Table 1:** Mass spectrometer compound parameters for cannabinoids and internal standard (IS)

Cannabinoid	Mass transition (***m/z***)	Cone voltage (V)	Collision energy (V)
**CBDV**	287.1 > 165.1	6	24
**THCV**	287.1 > 165.1	2	22
**CBN**	311.2 > 223.1	2	20
**CBC**	315.2 > 193.1	44	18
**CBD**	315.2 > 193.1	30	18
**Δ-8-THC**	315.2 > 193.1	26	22
**Δ-9-THC**	315.2 > 193.1	4	18
**CBL**	315.2 > 235.2	28	16
**CBG**	317.2 > 109.0	26	32
**Δ-9-THC-D3 (IS)**	318.3 > 196.1	72	24
**11-nor-9-COOH-Δ-9-THC-D9 (IS)**	352.1 > 194.3	68	26
**THCA**	356.9 > 245.1	4	30
**CBDA**	357.1 > 107.0	4	34
**CBGA**	359.2 > 136.0	36	32

All transitions (*m/z*) were selected and compound parameters optimized for each individual cannabinoid (*Δ-9-THC-D3* Δ-9-tetrahydrocannabinol-D3, *11-nor-9-COOH-Δ-9-THC-D9* 11-nor-9-carboxy-Δ-9-THC-D9, *CBDV* Cannabidivarin, *CBG* Cannabigerol, *CBD* Cannabidiol, *THCV* Tetrahydrocannabivarin, *CBN* Cannabinol, *Δ-9-THC* Δ-9-tetrahydrocannabinol, *Δ-8-THC* Δ-8-tetrahydrocannabinol, *CBL* Cannabicyclol, *CBC* Cannabichromene, *CBDA* Cannabidiolic acid, *CBGA* Cannabigerolic acid, and *THCA* Tetrahydrocannabinolic acid) using Intellistart™ feature of MassLynx® or by manual optimization, as necessary. (*V* voltage, *m/z* mass-to-charge ratio, *IS* internal standard)

For ESI^+^, the capillary voltage was 3.0 kV, the desolvation temperature was 450 °C, the desolvation gas flow was 800 L/h, and the cone gas flow was 60 L/h. For ESI^−^, the capillary voltage was -1.75 kV, the desolvation temperature was 450 °C, the desolvation gas flow was 650 L/h, and the cone gas flow was 50 L/h. MassLynx® 4.2 software was used to acquire the data and TargetLynx™ was used to quantify the amount of each cannabinoid (Waters, Milford, MA, USA).

### Preparation of calibration and quality control standards

Calibration standards (CS) were prepared from commercial stock solutions into two mix stocks of 5000 and 500 ng/mL of each cannabinoid in methanol. These mix stocks were then further diluted to provide calibration standards of 10, 50, 100, 150, 500, 1000, 1500, and 2500 ng/mL of each cannabinoid.

Quality control (QC) samples were prepared from the second set of mixed stocks to get final concentrations of 10, 75, 750, and 1750 ng/mL. Sample preparation of QCs used the same conditions as plant samples, which included vortex mixing, sonication, and centrifugation prior to analysis.

An IS stock was made at 500 ng/mL and added to CS, QC, and test samples to get a final concentration of 50 ng/mL.

Stock stability was assessed on the mix stock solutions after 6 months of storage at −20 °C. The mixed stock was used to prepare a standard curve while fresh QC samples of each individual cannabinoid were generated and quantified against the mixed stock curve.

### Sample preparation

Plant samples were dried in an oven at 55 °C for 72 h to ensure plant material was brittle. This time and temperature were chosen to minimize decarboxylation (Wang et al. 2016; Iffland et al. 2016). Samples were ground into a fine powder using a small coffee grinder. One of the two samples from the same plot was ground as the whole inflorescence with the stem and leaf included (top 15 cm) to obtain a composite sample, whereas the other one was trimmed, and only flowers were ground. For composite samples, the stem and leaves on average accounted for 9.4 ± 2.8% of the dried weight of the sample.

The dried, ground industrial hemp plant samples were carefully weighed in triplicate and cannabinoids were extracted by adding a solution of methanol and water (95:5, v/v) acidified with 0.005% formic acid. The plant material to solvent concentration ratio was 1:100 (w/v). After the addition of the extraction solvent, samples were vortex mixed for 5 min, sonicated for 5 min, and centrifuged at 4 °C, 3220×*g* for 10 min. Once spun down, the supernatant was serially diluted using a fresh extraction solvent to an appropriate final sample concentration to fall within the quantification range and meet range requirements.

### Analytical method validation

The method was validated for specificity, range, repeatability, reproducibility, and recovery in accordance with the International Council for Harmonization of Technical Requirements for Pharmaceuticals for Human Use (ICH) Q2(R1) Guidelines for analytical procedure validation (Validation of Analytical Procedures: Text and Methodology 2001). In addition, the Association of Official Analytical Chemists Standard Method Performance Requirements (AOAC SMPR) 2019.003 for quantification of cannabinoids in low THC varieties of hemp plant material was also followed (Standard Method Performance Requirements (SMPRs) for Quantification of cannabinoids in plant materials of hemp (Low THC Varieties Cannabis sp.) 2019).

### Application to mock regulatory study

The study was performed at the University of Florida’s North Florida Research and Education Center (NFREC) at Quincy, FL (30.54°N, 84.60°W) in 2019. The experimental design was a randomized complete block design with 4 replications. The seeds of ChBL, CT1, and CW were sown in the greenhouse into 128-cell seedling trays filled with PRO-MIX HP growth medium (Premier Horticulture Inc., Quakertown, PA, USA) on June 14, 2019. Seedlings were grown under supplemental lighting (16-h light and 8-h dark) to maintain vegetative growth. Irrigation was supplied as needed using overhead irrigation. Uniform seedlings of each variety were transplanted to the field on July 3, 2019. The field was set up with 20-cm high raised beds covered with plastic. Irrigation was supplied daily using drip tapes. Fertilizer (N-P_2_O_5_-K_2_O: 10-10-10) was applied at a rate of 112 kg ha^−1^ immediately prior to transplanting and disked into soils. A soluble fertilizer (N-P_2_O_5_-K_2_O: 4-0-8) was applied with irrigation as needed throughout the season based on an accumulated rate of 56 kg N ha^−1^. Anthesis was observed on August 7, 2019, when the day-length was ~13.5 h. Two top 15 cm samples were taken on October 10, 2019 from 26 experimental plots resulting in 56 samples.

### Statistical analysis

R Studio version 3.6.0 was used for statistical analysis (R Foundation for Statistical Computing, Vienna, Austria). A two-tailed paired *t* test was performed for each cannabinoid to analyze if a difference existed between the sampling method (flower vs composite) at a significance level of α ≤ 0.05. A two-way ANOVA was performed to determine the effect of variety and treatment on cannabinoid levels and if there existed any interaction between the factors: sample type and variety. Additionally, the agreement between sets was evaluated by calculating the intraclass correlation coefficient. For CBD, CBG, and Δ-9-THC, the neutral and acidic forms were added together using the following formula to obtain the total cannabinoid content to be used in statistical analyses:

$$ Total\ content={Concentration}_{neutral}+\left({Concentration}_{acid}\ast 0.877\right) $$

## Results

### UPLC-MS/MS method development and validation

**Fig. 2:**
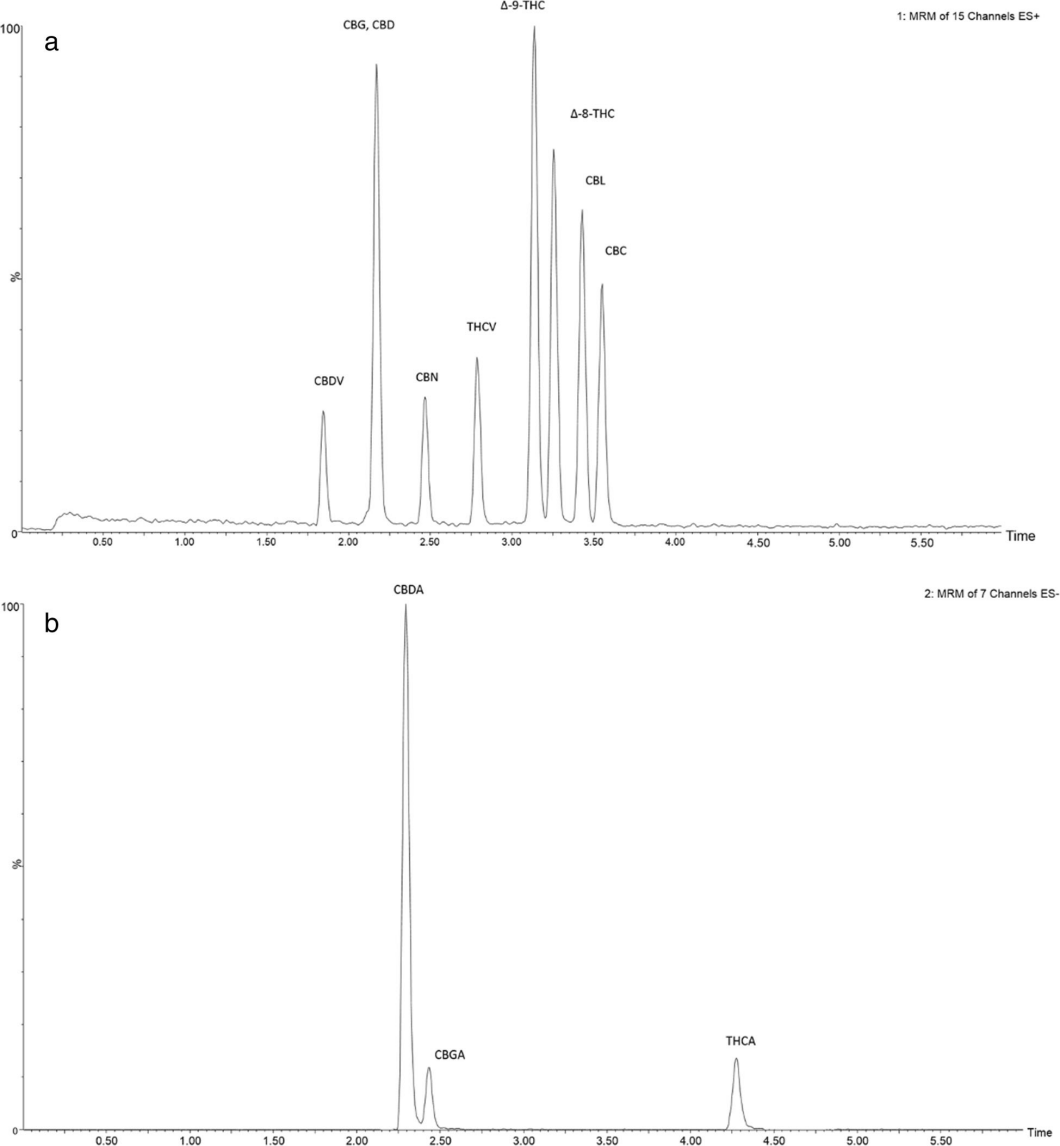
Representative chromatograms. Representative chromatograms of standard cannabinoid concentrations (100 ng/mL, each) in **a** positive and **b** negative ionization mode. Retention times for each cannabinoid are as follows: Cannabidivarin (CBDV)—1.9 min, Cannabigerol (CBG)—2.1 min, Cannabidiol (CBD)—2.2 min, Tetrahydrocannabivarin (THCV)—2.5 min, Cannabinol (CBN)—2.8 min, Δ-9-tetrahydrocannabinol (Δ-9-THC)—3.2 min, Δ-8-tetrahydrocannabinol (Δ-8-THC)—3.3 min, Cannabicyclol (CBL)—3.4 min, Cannabichromene (CBC)—3.6 min, Cannabidiolic acid (CBDA)—2.3 min, Cannabigerolic acid (CBGA)—2.5 min, and Tetrahydrocannabinolic acid (THCA)—4.3 min

A rapid and reliable method was developed for the quantification of 12 cannabinoids in hemp samples. Representative chromatograms for both positive and negative ionization modes at 100 ng/mL are shown in Fig. 2.

### Specificity

The method was validated for specificity by separating four compounds with the same molecular weight (CBC, CBD, Δ-8-THC, Δ-9-THC, CBL) which can be seen baseline separated in Fig. 2.

### Calibration range, linearity, and stock stability

The recommended ranges according to the AOAC SMPR 2019.003 are 0.05 to 5% w/w for all cannabinoids except CBD and CBDA, which have a recommended range of 0.05 to 35% w/w.

Based on the recommendations, a calibration range of 10–2500 ng/mL representing 0.05–35% w/w of cannabinoid content was selected. Linearity was seen over this range using a 1/x^2^ weighing method resulting in a correlation coefficient >0.99 for all cannabinoids. The concentration of 10 ng/mL was chosen as the limit of quantification for all cannabinoids as it always resulted in a signal to noise ratio of greater than 10:1. The limit of detection for this method was determined to be 1 ng/mL as it always resulted in a signal to noise ratio greater than 3:1 for all cannabinoids.

Freshly prepared QC samples were made for each individual alkaloid (75 and 1750 ng/mL, *N* = 3). These were then quantified using a curve generated from a mixed stock solution that had been stored at −20 °C for 6 months. Accuracy of the individual cannabinoids fell within 15% of the nominal concentration (85–115) at LQC and HQC when quantified against the mixed stock calibration curve. This indicates that cannabinoids do not degrade in mixed stock within 6 months. The results can be seen in Table 2.

**Table 2:** Stock solution stability results for each cannabinoid

**Concentration (ng/mL)**	**Cannabinoid**					
	**CBC** **% Nominal ± SD**	**CBD** **% Nominal ± SD**	**CBDA** **% Nominal ± SD**	**CBDV** **% Nominal ± SD**	**CBG** **% Nominal ± SD**	**CBGA** **% Nominal ± SD**
75	98.2 ± 11.9	94.1 ± 1.3	99.4 ± 1.5	93.7 ± 3.0	96.7 ± 6.0	96.9 ± 3.0
1750	102.8 ± 4.5	109.3 ± 2.3	99.9 ± 13.8	100.3 ± 7.9	91.6 ± 0.7	103.1 ± 0.3
**Concentration (ng/mL)**	**Cannabinoid**					
	**CBL** **% Nominal ± SD**	**CBN** **% Nominal ± SD**	**Δ-8-THC** **% Nominal ± SD**	**Δ-9-THC** **% Nominal ± SD**	**THCA** **% Nominal ± SD**	**THCV** **% Nominal ± SD**
75	108.4 ± 4.3	108.1 ± 5.9	98.6 ± 6.0	92.0 ± 1.9	95.1 ± 8.2	93.5 ± 4.4
1750	101.2 ± 5.3	108.2 ± 0.4	99.0 ± 2.9	97.8 ± 1.1	94.8 ± 7.9	93.0 ± 3.3

Mixed stocks were stored at −20 °C for 6 months. Individual stocks were freshly prepared and % nominal was calculated as observed concentration/nominal concentration *100 from the mixed stock generated curve. All values represent as mean ± standard deviation (*N* = 3)

*CBDV* Cannabidivarin, *CBG* Cannabigerol, *CBD* Cannabidiol, *THCV* Tetrahydrocannabivarin, *CBN* Cannabinol, *Δ-9-THC* Δ-9-tetrahydrocannabinol, *Δ-8-THC* Δ-8-tetrahydrocannabinol, *CBL* Cannabicyclol, *CBC* Cannabichromene, *CBDA* Cannabidiolic acid, *CBGA* Cannabigerolic acid, and *THCA* Tetrahydrocannabinolic acid

### Repeatability, reproducibility, and recovery

Over a period of 3 days, six replicates at four concentrations (10, 75, 750, and 1750 ng/mL) were analyzed to determine the repeatability (intra-day) and reproducibility (inter-day) of the method. The accuracy and precision for intra- and inter-day samples for each individual cannabinoid can be seen in Tables 3 and 4. Precision was measured as the percent relative standard deviation which was calculated by multiplying the standard deviation by 100 then dividing this value by the mean. Accuracy was measured as percent bias which was calculated by subtracting the observed mean from the nominal concentration then dividing this value by the nominal concentration prior to multiplying by 100 to get the percent bias. For repeatability, the percent relative standard deviation values were always ≤5% at 0.05% w/w, ≤3% in the 0.05–5% w/w range, and ≤2% for the 5–35% w/w range.

**Table 3:** Intra-day accuracy and precision for cannabinoids of the assay method. The results verify the repeatability of the assay method as required by AOAC SMRP 2019.003 (Standard Method Performance Requirements (SMPRs) for Quantification of cannabinoids in plant materials of hemp (Low THC Varieties Cannabis sp.) 2019)

**Concentration (ng/mL)**	**Cannabinoid**								
	**CBC**			**CBD**			**CBDA**		
	**Measured Concentration (mean ± SD)**	**Precision (%RSD)**	**Accuracy (% bias)**	**Measured Concentration (mean ± SD)**	**Precision (%RSD)**	**Accuracy (% bias)**	**Measured Concentration (mean ± SD)**	**Precision (%RSD)**	**Accuracy (% bias)**
10	10.3 ± 0.5	4.6	2.7	10.3 ± 0.5	4.9	3.2	10.5 ± 0.3	3.3	4.8
75	72.3 ± 0.9	1.3	−3.7	72.3 ± 1.4	1.9	−3.6	74.2 ± 2.0	2.7	−1.1
750	768.6 ± 18.7	2.4	2.5	755.6 ± 17.6	2.3	0.7	739.6 ± 20.5	2.8	−1.4
1750	1856.8 ± 31.8	1.7	6.1	1842.8 ± 30.2	1.6	5.3	1760.9 ± 33.8	1.9	0.6
**Concentration (ng/mL)**	**Cannabinoid**								
	**CBDV**			**CBG**			**CBGA**		
	**Measured Concentration (mean ± SD)**	**Precision (%RSD)**	**Accuracy (% Bias)**	**Measured Concentration (mean ± SD)**	**Precision (%RSD)**	**Accuracy (% Bias)**	**Measured Concentration (mean ± SD)**	**Precision (%RSD)**	**Accuracy (% Bias)**
10	9.2 ± 0.2	2.2	−8.4	11.0 ± 0.4	3.4	10.5	10.5 ± 0.5	4.9	5.1
75	73.2 ± 1.4	1.9	−2.3	69.0 ± 2.2	3.2	−8.1	72.1 ± 0.6	0.9	−3.9
750	767.8 ± 18.6	2.4	2.4	760.2 ± 17.2	2.3	1.4	732.7 ± 15.9	2.2	−2.3
1750	1826.8 ± 26.8	1.5	4.4	1851.1 ± 29.3	1.6	5.8	1754.8 ± 48.6	2.8	0.3
**Concentration (ng/mL)**	**Cannabinoid**								
	**CBL**			**CBN**			**Δ-8-THC**		
	**Measured Concentration (mean ± SD)**	**Precision (%RSD)**	**Accuracy (% Bias)**	**Measured Concentration (mean ± SD)**	**Precision (%RSD)**	**Accuracy (% Bias)**	**Measured Concentration (mean ± SD)**	**Precision (%RSD)**	**Accuracy (% Bias)**
10	10.3 ± 0.4	3.5	2.6	10.1 ± 0.5	4.6	1.3	10.6 ± 0.4	3.6	5.6
75	76.4 ± 1.4	1.8	1.9	79.1 ± 2.2	2.8	5.4	79.2 ± 1.7	2.1	5.6
750	786.9 ± 16.8	2.1	4.9	757.0 ± 18.1	2.4	0.9	792.9 ± 16.4	2.1	5.7
1750	1833.8 ± 25.0	1.4	4.8	1732.9 ± 13.7	0.8	−1.0	1784.6 ± 23.9	1.3	2.0
**Concentration (ng/mL)**	**Cannabinoid**								
	**Δ-9-THC**			**THCA**			**THCV**		
	**Measured Concentration (mean ± SD)**	**Precision (%RSD)**	**Accuracy (% Bias)**	**Measured Concentration (mean ± SD)**	**Precision (%RSD)**	**Accuracy (% Bias)**	**Measured Concentration (mean ± SD)**	**Precision (%RSD)**	**Accuracy (% Bias)**
10	9.8 ± 0.3	2.9	−1.5	10.3 ± 0.5	4.0	3.4	9.9 ± 0.4	5.5	−0.9
75	79.7 ± 1.7	1.9	6.3	77.6 ± 1.6	2.1	3.5	79.9 ± 1.8	2.3	6.6
750	753.8 ± 16.4	1.3	0.5	710.8 ± 15.5	2.2	−5.2	771.8 ± 14.8	1.9	2.9
1750	1760.7 ± 23.9	0.8	0.6	1625.1 ± 24.0	1.5	−7.1	1763.4 ± 25.6	1.5	0.8

Precision was measured as the percent relative standard deviation which was calculated by multiplying the standard deviation by 100 then dividing this value by the mean. Accuracy was measured as percent bias which was calculated by subtracting the observed mean from the nominal concentration then dividing this value by the nominal concentration prior to multiplying by 100 to get the percent bias

SD Standard deviation, *%RSD* Percent relative standard deviation, *CBDV* Cannabidivarin, *CBG* Cannabigerol, *CBD* Cannabidiol, *THCV* Tetrahydrocannabivarin, *CBN* Cannabinol, *Δ-9-THC* Δ-9-tetrahydrocannabinol, *Δ-8-THC* Δ-8-tetrahydrocannabinol, *CBL* Cannabicyclol, *CBC* Cannabichromene, *CBDA* Cannabidiolic acid, *CBGA* Cannabigerolic acid, and *THCA* Tetrahydrocannabinolic acid

**Table 4:** Inter-day accuracy and precision for cannabinoids of the assay method. The results verify the reproducibility of the assay method as required by AOAC SMRP 2019.003 (Standard Method Performance Requirements (SMPRs) for Quantification of cannabinoids in plant materials of hemp (Low THC Varieties Cannabis sp.) 2019)

**Concentration (ng/mL)**	**Cannabinoid**								
	**CBC**			**CBD**			**CBDA**		
	**Measured Concentration (mean ± SD)**	**Precision (%RSD)**	**Accuracy (% Bias)**	**Measured Concentration (mean ± SD)**	**Precision (%RSD)**	**Accuracy (% Bias)**	**Measured Concentration (mean ± SD)**	**Precision (%RSD)**	**Accuracy (% Bias)**
10	10.3 ± 0.4	4.1	3.2	10.2 ± 0.4	4.0	1.8	10.4 ± 0.6	5.3	4.1
75	72.5 ± 4.6	6.4	−3.3	72.5 ± 2.4	3.3	−3.3	76.3 ± 4.7	6.1	1.7
750	760.1 ± 28.1	3.7	1.3	749.9 ± 21.4	2.9	0.0	739.0 ± 30.5	4.1	−1.5
1750	1868.4 ± 58.4	3.1	6.8	1841.3 ± 48.9	2.7	5.2	1734.9 ± 76.7	4.4	−0.9
**Concentration (ng/mL)**	**Cannabinoid**								
	**CBDV**			**CBG**			**CBGA**		
	**Measured Concentration (mean ± SD)**	**Precision (%RSD)**	**Accuracy (% Bias)**	**Measured Concentration (mean ± SD)**	**Precision (%RSD)**	**Accuracy (% Bias)**	**Measured Concentration (mean ± SD)**	**Precision (%RSD)**	**Accuracy (% Bias)**
10	9.6 ± 0.6	6.0	−3.8	10.3 ± 0.8	8.1	2.7	10.6 ± 0.9	8.2	5.7
75	73.6 ± 3.6	4.8	−1.8	70.5 ± 3.7	5.3	−-6.0	74.2 ± 4.2	5.7	−1.0
750	765.8 ± 27.8	3.6	2.1	755.6 ± 24.9	3.3	0.7	735.0 ± 19.8	2.7	−2.0
1750	1852.4 ± 56.3	3.0	5.9	1880.0 ± 58.9	3.1	7.4	1731.0 ± 77.0	4.4	−1.1
**Concentration (ng/mL)**	**Cannabinoid**								
	**CBL**			**CBN**			**Δ-8-THC**		
	**Measured Concentration (mean ± SD)**	**Precision (%RSD)**	**Accuracy (% Bias)**	**Measured Concentration (mean ± SD)**	**Precision (%RSD)**	**Accuracy (% Bias)**	**Measured Concentration (mean ± SD)**	**Precision (%RSD)**	**Accuracy (% Bias)**
10	10.3 ± 0.5	4.4	3.0	9.8 ± 0.4	4.3	−1.8	10.3 ± 0.4	4.3	3.3
75	74.5 ± 4.6	6.2	-0.7	76.8 ± 4.0	5.1	2.4	75.4 ± 5.0	6.7	0.5
750	779.6 ± 25.6	3.3	4.0	755.9 ± 29.2	3.9	0.8	778.8 ± 35.5	4.6	3.8
1750	1877.3 ± 67.3	3.6	7.3	1736.0 ± 55.0	3.2	−0.8	1762.2 ± 52.5	3.0	0.7
**Concentration (ng/mL)**	**Cannabinoid**								
	**Δ-9-THC**			**THCA**			**THCV**		
	**Measured Concentration (mean ± SD)**	**Precision (%RSD)**	**Accuracy (% Bias)**	**Measured Concentration (mean ± SD)**	**Precision (%RSD)**	**Accuracy (% Bias)**	**Measured Concentration (mean ± SD)**	**Precision (%RSD)**	**Accuracy (% Bias)**
10	9.8 ± 0.6	5.9	−1.6	9.8 ± 0.7	6.8	−1.9	10.0 ± 0.4	4.4	0.0
75	77.9 ± 3.3	4.2	3.8	79.0 ± 4.1	5.2	5.3	78.3 ± 3.4	4.3	4.3
750	758.2 ± 24.8	3.3	1.1	719.7 ± 34.1	4.7	−4.0	760.4 ± 33.1	4.4	1.4
1750	1741.8 ± 64.9	3.7	−0.5	1621.6 ± 100.2	6.2	−7.3	1738.6 ± 59.1	3.4	−0.6

Precision was measured as the percent relative standard deviation which was calculated by multiplying the standard deviation by 100 then dividing this value by the mean. Accuracy was measured as percent bias which was calculated by subtracting the observed mean from the nominal concentration then dividing this value by the nominal concentration prior to multiplying by 100 to get the percent bias

*SD* Standard deviation, *%RSD* Percent relative standard deviation, *CBDV* Cannabidivarin, *CBG* Cannabigerol, *CBD* Cannabidiol, *THCV* Tetrahydrocannabivarin, *CBN* Cannabinol, *Δ-9-THC* Δ-9-tetrahydrocannabinol, *Δ-8-THC* Δ-8-tetrahydrocannabinol, *CBL* Cannabicyclol, *CBC* Cannabichromene, *CBDA* Cannabidiolic acid, *CBGA* Cannabigerolic acid, and *THCA* Tetrahydrocannabinolic acid

For reproducibility, the relative standard deviation fell within ≤10% at 0.05% w/w, ≤8% in the 0.05–5% w/w range, and ≤6% for the 5–35% w/w range.

Recovery was measured by spiking dried *Humulus lupulus* plant samples, used because they come from the same taxonomical family as cannabis, Cannabaceae, with a known quantity of cannabinoids. These samples were then prepared in the exact same way as an analytical sample and ran through the UPLC-MS/MS method to determine recovery percentage. Recovery was calculated by dividing the observed concentration by the nominal concentration and multiplying this value by 100. The recovery percentages are shown in Table 5 and all were within the ranges recommended by AOAC SMPR 2019.003.

**Table 5:** Percent recovery study results recovery was calculated as Observed Concentration/Nominal Concentration *100. Data represented as mean **±** standard deviation (SD)

**Concentration (ng/mL)**	**Cannabinoid**					
	**CBC**	**CBD**	**CBDA**	**CBDV**	**CBG**	**CBGA**
	**Mean % Recovery ± SD**	**Mean % Recovery ± SD**	**Mean % Recovery ± SD**	**Mean % Recovery ± SD**	**Mean % Recovery ± SD**	**Mean % Recovery ± SD**
10	115.0 ± 0.8	100.6 ± 1.5	112.4 ± 2.1	117.9 ± 1.2	114.1 ± 2.3	98.4 ± 1.5
75	108.6 ± 0.5	106.4 ± 0.2	109.7 ± 3.5	106.9 ± 0.1	106.2 ± 1.9	101.2 ± 4.3
750	103.3 ± 2.2	102.3 ± 2.4	100.5 ± 1.6	101.5 ± 2.6	101.6 ± 1.7	98.0 ± 0.3
1750	106.3 ± 1.5	103.2 ± 1.7	100.3 ± 4.0	104.3 ± 1.7	105.1 ± 1.0	102.8 ± 1.1
**Concentration (ng/mL)**	**Cannabinoid**					
	**CBL**	**CBN**	**Δ-8-THC**	**Δ-9-THC**	**THCA**	**THCV**
	**Mean % Recovery ± SD**	**Mean % Recovery ± SD**	**Mean % Recovery ± SD**	**Mean % Recovery ± SD**	**Mean % Recovery ± SD**	**Mean % Recovery ± SD**
10	110.1 ± 1.7	104.6 ± 1.7	106.3 ± 3.4	107.1 ± 2.9	108.0 ± 3.4	105.1 ± 1.8
75	108.8 ± 0.6	110.6 ± 0.1	110.5 ± 1.8	107.3 ± 1.2	110.1 ± 1.5	108.6 ± 1.1
750	103.7 ± 1.6	101.9 ± 1.9	106.0 ± 1.6	101.1 ± 1.4	98.5 ± 1.4	101.2 ± 1.3
1750	104.5 ± 1.5	100.7 ± 1.7	104.5 ± 1.9	101.1 ± 0.2	100.2 ± 1.8	101.3 ± 1.8

*CBDV* Cannabidivarin, *CBG* Cannabigerol, *CBD* Cannabidiol, *THCV* Tetrahydrocannabivarin, *CBN* Cannabinol, *Δ-9-THC* Δ-9-tetrahydrocannabinol, *Δ-8-THC* Δ-8-tetrahydrocannabinol, *CBL* Cannabicyclol, *CBC* Cannabichromene, *CBDA* Cannabidiolic acid, *CBGA* Cannabigerolic acid, and *THCA* Tetrahydrocannabinolic acid

### Uncertainty

The uncertainty for each cannabinoid at each concentration level can be calculated using the formula U = k * RSD provided by the FDA Office of Regulatory Affairs (ORA Laboratory Manual 2019). The relative standard deviation used in this calculation was the one generated from the inter-day validation. The coverage factor at 95%, *k*, for *N* = 18 would be 2.11. Therefore, for Δ-9-THC near the threshold for legality, the uncertainty is 8.86% or ±0.03. When reporting values for regulatory purposes, the concentration of the cannabinoid is presented with the uncertainty limit added as the standard deviation.

### Study results

The full cannabinoid profile was obtained for 56 plant samples. The major cannabinoids present in all samples were CBC, CBD, CBDA, CBG, CBGA, Δ-9-THC, and THCA. All other cannabinoids were below the limit of quantification (≤0.05% w/w).

A two-tailed paired *t* test for total CBG and CBC gave a result of no significant difference between flower and composite samples. Alternatively, the results of the paired *t* test for total THC and total CBD indicated a significant difference of 0.09 and 1.32% between flower and composite samples, respectively. The intraclass correlation coefficient for each set of tests was also calculated. For total THC and total CBD, there was a poor agreement between the sets while total CBG had a moderate agreement and CBC had a good agreement between tests, providing further assurance that the measured difference in groups was valid (Table 6).

**Table 6:** Statistical analysis of CBC, total CBD, total CBG, and total THC in flower versus composite samples

Cannabinoid	Sample type		Mean difference	Intraclass correlation coefficient
	Flower (% w/w)	Composite (% w/w)		
**CBC**	0.27 ± 0.19	0.25 ± 0.19	0.02	0.79
**Total CBD**	12.3 ± 2.51	11.0 ± 1.98	1.32*	0.32
**Total CBG**	0.28 ± 0.11	0.25 ± 0.11	0.03	0.55
**Total THC**	0.69 ± 0.16	0.60 ± 0.13	0.09*	0.29

Values represent the mean ± standard deviation. A two-tailed paired *t* test was performed to determine if there was a significant mean difference between the composite and flower only samples. *indicates a significant difference at *p* ≤ 0.05. Intraclass correlation coefficient was also calculated for each group to determine the degree to which values from the same group agree. This coefficient is interpreted as follows: <0.5 poor agreement, 0.5–0.75 moderate agreement, 0.75–0.9 good agreement, and >0.9 excellent agreement

*CBC* Cannabichromene, *CBD* Cannabidiol, *CBG* Cannabigerol, *THC* Tetrahydrocannabinol

Further, the individual varieties were examined to investigate if variation existed between the variety for the sample type. A two-way ANOVA was performed investigating the combined effect of sample type and variety. For both total CBD and total THC, there was no significant difference in the means of the interaction of the two factors, with *p* values of 0.31 and 0.38, respectively.

## Discussion

The method developed was validated for the analysis of industrial hemp samples and determined to be rapid, reliable, and robust. The method had a short run time of 6 minutes which did not allow for CBD and CBG to be separated chromatographically but this was simply solved with mass detection of unique fragmentation patterns attributable to each cannabinoid. Cannabinoid assay methods available in the literature to simultaneously quantify over ten cannabinoids were 8 minutes or more, so the short run time of the developed method will greatly improve throughput for laboratories analyzing hemp for regulatory purposes.

As an LC method, the acidic and neutral cannabinoids are quantified individually and total THC must be calculated after analysis. For this project, that was important in order to be able to investigate and define the concentration of acidic and neutral compounds separately in the plant over time (Yang et al. 2020). But in the regulatory setting, where only total THC needs to be reported, GC methods will calculate this value in the detector because as the sample is heated the acidic compounds are converted to their neutral form.

When considering detection methods, MS is sensitive and selective which is ideal when monitoring many compounds that are similar in structure and mass, as is the case with phytocannabinoids in hemp (Nie et al. 2019), but thus requires samples to be diluted extensively prior to analysis. This method can detect cannabinoids at a level of 0.005% on a dry weight basis. Other detection systems, such as UV or DAD are not as sensitive and selective but allow for higher concentrations of analytes to be injected for analysis which may decrease sample preparation time (Wang et al. 2016; Vaclavik et al. 2019; Zivovinovic et al. 2018).

Other countries have designated standard equipment and methods for the determination of total THC in industrial hemp (Industrial Hemp Technical Manual 2004). As it stands, the United States has not selected a standard method but studies have already indicated that cannabinoid test results are inconsistent between laboratories (Jikomes and Zoorob 2018). Therefore, it is imperative that standard methods be suggested to decrease the potential for variation between results.

In addition to the variability that may exist between laboratories and testing methods, there is also the potential for variability when considering how plants are sampled. The results of this study show that there is the potential for significant differences in cannabinoid content based on which plant part is sampled. A decrease in 0.09% w/w of total THC was seen between a flower sample and a 15-cm composite sample. As the margin for error when it comes to a crop being legal or illegal at the federal level is very slim, these results are important to consider when drafting sampling guidelines for industrial hemp crops. If the process of sampling is not standardized, the same crop could test above or below the legal threshold based on the manner in which the crop was sampled. In this study, only one length (15 cm) was investigated, so future studies would consider various lengths to see how to dilute a flower sample becomes as more leaf and stem biomass is added. Environmental factors such as soil quality, geographical location, temperature, and rainfall, among others, could also be influential in the development of cannabinoids so only sampling 26 plants grown in the same area is insufficient. Further studies could examine plants grown in various regions to determine if the difference between flower only and composite samples prevails. Also, this study only examined three CBD-type hemp varieties, but in the future, this research could be expanded to include fiber, grain, and dual-purpose industrial hemp varieties.

Though sample analysis and sample type differences may seem insignificant when considered individually, when combined, there is the possibility of significant legal and economic ramifications.

## Conclusions

From a regulatory perspective, these results indicate that the way industrial hemp samples are taken and analyzed may influence the legality of a crop. To determine the relative difference, the percent change was calculated using the formula:

$$ Percent\ Change=\left( Mean\ difference/ Overall\ mean\right)\ast 100 $$

For total CBD, the percent change was 11% and for total THC the percent change was 14% between sampling types. When this is added to the uncertainty of the method, which was calculated to be 9%, there is an opportunity for a 23% difference in total THC. This has the potential to influence whether crop tests as industrial hemp or cannabis. As any industrial hemp crop testing over the legal limit must be destroyed, the consequences of having a significant deal of variation in sampling and analysis are substantial. When considering the many factors involved that could influence the testing results for industrial hemp and with the threshold for legality being so low, descriptive and strict sampling and testing methods must be defined in order to standardize and achieve consistent results.

## Data Availability

The datasets used and/or analyzed during the current study are available from the corresponding author on reasonable request.

## Abbreviations

11-nor-9-COOH-Δ-9-THC-D9: 11-nor-9-carboxy-Δ-9-THC-D9
Δ-8-THC: Delta-8-tetrahydrocannabinol;
Δ-9-THC: Delta-9-tetrahydrocannabinol
Δ-9-THC-D3: Delta-9-tetrahydrocannabinol-D3
AOAC SMPR: Association of Official Analytical Chemists Standard Method Performance Requirements
CBC: Cannabichromene
CBD: Cannabidiol
CBDA: Cannabidiolic acid
CBDV: Cannabidivarin
CBG: Cannabigerol
CBGA: Cannabigerolic acid
CBL: Cannabicyclol
CBN: Cannabinol
ChBL: Cherry Blossom
CS: Calibration standards
CT1: Cherry x T1
CW: Cherry Wine
GC: Gas chromatography
ICH: International Council for Harmonization
IS: Internal Standard
LC: Liquid chromatography
QC: Quality control
THCA: Δ-9-Tetrahydrocannabinolic acid
THCV: Tetrahydrocannabivarin
UF/IFAS: University of Florida’s Institute of Food and Agricultural Science
UPLC-MS/MS: Ultra-performance liquid chromatography-tandem mass spectrometry
USDA: United States Department of Agriculture
